# Exploring the influence of probiotic administration routes on immune responses in atopic march

**DOI:** 10.3389/fimmu.2025.1601596

**Published:** 2025-08-01

**Authors:** Fang-Yu Zhang, Chi-Yu Yang, Chien-Hsun Huang, I-Jen Wang

**Affiliations:** ^1^ Animal Technology Research Center, Agriculture Technology Research Institute, Miaoli, Taiwan; ^2^ Bioresource Collection and Research Center (BCRC), Food Industry Research and Development Institute, Hsinchu, Taiwan; ^3^ Department of Pediatrics, Taipei Hospital, Ministry of Health and Welfare, Taipei, Taiwan; ^4^ School of Medicine, National Yang Ming Chiao Tung University, Taipei, Taiwan; ^5^ College of Public Health, China Medical University, Taichung, Taiwan; ^6^ National Institute of Environmental Health Sciences, National Health Research Institutes, Miaoli, Taiwan

**Keywords:** atopic dermatitis, asthma, probiotics, nasal administration, atopic march

## Abstract

**Background:**

Children with atopic dermatitis (AD) have a higher likelihood of developing asthma, the so-called atopic march. Previous studies have suggested that probiotics can modulate development of the immune system and atopic disorders. However, the exact mechanisms and whether the route of administration of probiotics has a clinical effect are unknown. Therefore, we conducted this study to investigate whether different routes of administration of probiotics may have different effects.

**Method:**

Probiotics Bacteroides plebeius, B. ovatus, 74-B and YCFA-33 were administered to mice via oral and nasal routes for 4 weeks, followed by the induction of AD using ovalbumin (OVA). The condition of the stimulated skin and histology of skin tissues were evaluated. In addition, 3 days of consecutive exposure to OVA (3%) aerosol was used to induce asthma at the end of the AD experiment. Serum immunoglobulin E (IgE), IgG, IL-4, IFN-γ, and TNF-α levels and histological evaluations of lung tissues were assessed after the experiments.

**Result:**

The oral administration of probiotics B. plebeius and B. ovatus may have improved the inflammatory response of OVA-induced asthma. The nasal administration of the probiotics 74-B and YCFA-33 may have alleviated the symptoms of skin redness and itching of OVA-induced atopic dermatitis. These effects may have been due to reduced infiltration of white blood cells in the stimulated skin area and dampened inflammatory responses. In the later asthma model, YCFA-33 administration significantly increased total IgG and IgG1 in serum, reduced OVA-IgE levels and IL-4 levels, decreased neutrophil content and TNF-α expression, and increased IFN-γ levels in lung lavage fluid (p<0.05). These effects may have blocked the progression from AD to asthma pulmonary inflammation.

**Conclusion:**

B. ovatus had better effects via oral administration while 74-B and YCFA-33 had better effects via nasal administration. Oral administration is not always the best route. Probiotics may mitigate allergic reactions and pulmonary inflammation. These findings could contribute to the development of innovative biomarkers and early interventions for managing asthma and atopic disorders.

## 1 Introduction

Recent advances in microbiome research have shed light on the pivotal role of gut commensal bacteria in modulating immune responses, particularly in the context of allergic diseases. Among these, Bacteroides plebeius, Bacteroides ovatus, Faecalibacterium duncaniae, and Faecalibacterium prausnitzii have drawn increasing interest due to their immunomodulatory properties and potential as biomarkers of allergic disease severity. B. plebeius, traditionally noted for its presence in individuals consuming seaweed-rich diets, has been identified in lower abundance among children with food allergies, suggesting a possible protective role (1). Similarly, B. ovatus, a common human gut symbiont, has demonstrated capacity to induce regulatory T cells and promote epithelial barrier integrity, both of which are crucial in preventing allergen sensitization (2). On the other hand, Faecalibacterium duncaniae and its close relative F. prausnitzii, both key butyrate-producing bacteria, are widely regarded as anti-inflammatory microbes. Clinical studies have reported reduced levels of F. prausnitzii in patients with atopic dermatitis and asthma, correlating with elevated inflammatory cytokines and increased allergic symptoms (3). The recent reclassification of F. prausnitzii strains into F. duncaniae provides further resolution in identifying microbial signatures associated with health and disease states (4). Collectively, these bacteria represent promising microbial indicators and potential therapeutic targets for modulating allergic inflammation. Understanding their roles and interactions with the host immune system could enable the development of microbiota-targeted interventions to prevent or treat allergic diseases (5).

## 2 Methods

### 2.1 Animal experiment

Six to eight-week-old female BALB/c mice were used in the experiments. These mice were purchased from National Laboratory Animal Center (Taiwan). Animal care and all experimental procedures were approved and conducted in accordance with the guidelines of the Institutional Animal Care and Use Committee of the Agricultural Technology Research Institute (Approval number: IACUC No111048). Raised in an IVC independent ventilation cage system, 5 animals per cage, managed by dedicated personnel. The constant temperature of 20~26°C and 30%-70% humidity was maintained in the breeding environment with a light-to-dark cycle of 12:12 h. After one week of acclimation, the 60 mice were used and randomly divided into six groups (ten mice per group) as follows: untreated group (normal control, C), negative control group (OVA-sensitized group), Bateroides plebeius (Group B.P), B. ovatus (Group B.O), 74-B(Group 74B) and YCFA-33 (YCFA-33 group). For more information, see **Table 1**. Before administration of the test substance, there should be no significant difference (p>0.05) in the average body weight between groups and between each group.

**Table 1:** Animal Group.

Group	Intranasal administration	OVA induction	Skin induction	Asthma induction	count
Normal group	0.04 mL/mice	None	Saline 0.1mL/mice	Saline 0.04mL/mice	10
Negative control group		(20 μg/0.2 mL/mice, i.p)	OVA (100 μg/mL) 0.1mL/mice	3% OVA 0.04mL/mice	10
Bateroides plebeius (Group B.P)					10
B. ovatus (Group B.O)					10
74B (Group 74B)					10
YCFA-33 (YCFA-33 group)					10
**Total**					**60**

Mice were given a dose of 0.04 mL/mice by nasal inhalation once a day for 73 consecutive days. After anesthetizing the mice with 4-5% isoflurane, they were secured in a restrained manner using one hand. Using a micropipette, 0.04 mL of the control/test substance was slowly instilled into the mouse’s nostrils. The process was carried out slowly to prevent respiratory distress in the animals. The normal group and negative control group mice received intranasal administration of 0.04 mL/mice of physiological saline. The experimental group mice received intranasal administration of 0.04 mL/mice of the test substance.

### 2.2 Induction of atopic dermatitis

The mice in the group without C were intraperitoneally inoculated with OVA (20 μg/200 μL/mice) (grade V; Sigma, MO, USA) mixed with 4 mg of aluminum hydroxide (ImjectAlum; Thermo Fisher Scientific, MA, USA) in a volume of 0.2mL three times at one-week intervals (i.e., on days 0, 7, and 14). The mice were anesthetized by isoflurane inhalation using electric clippers specially designed for small animals to remove hair roughly, and then use depilatory cream to remove hair in detail. The OVA patches were prepared with 1×1 cm2 with 100 μg OVA in phosphate-buffered saline (PBS). The OVA patches were attached on the shaved dorsal skin with a Tegaderm (3M, USA) for seven days (i.e., from days 14 to 20) and were changed daily. On the 36th day, OVA (100μg/100μL/mice) was applied to the skin surface for local stimulation induction, and normal saline was applied to the normal group. After 1 week of stasis after intraperitoneal injection, OVA was used to stimulate the skin for 7 days. This is one cycle, with a total of 3 cycles. Method for inducing asthma mode in the late stage of atopic dermatitis: 73 days after AD mode was induced, asthma mode was induced with 3% OVA aerosol for 3 consecutive days.

### 2.3 Blood collection and analysis

Cardiac blood was collected after inducing anesthesia. Blood was drawn into gel tubes, left to stand for 30 minutes, and then centrifuged at 3,500 rpm for 15 minutes. The supernatant was collected and stored at -70°C for subsequent analysis of Total IgG, IgE, IgG1, and specific IgE content in the serum.

### 2.4 Bronchoalveolar lavage fluid collection

Lungs were lavaged three times with 0.5mL PBS, collecting at least 1mL of BALF. The collected fluid was centrifuged at 2000 rpm, 4°C for 15 minutes. The supernatant was stored at -80°C for analysis of IL-4, IFN-γ, and IL-5. The cellular layer was treated with sterile PBS to disperse the cells. Red blood cells were lysed using RBC lysis leaving only white blood cells, which were then analyzed using a hematology analyzer (ProCyte Dx, IDEXX) to determine cell types and counts. Evaluate leukocyte parameters: White Blood Cell (WBC) count, Neutrophils (NEU), Lymphocytes (LYM; count and percentage), Monocytes (MONO), Eosinophils (EOS), Basophils (BASO), Bands, Agranulocytes (AGRANS), and Granulocytes (GRANS).

### 2.5 Measurement of clinical severity

After skin stimulation with OVA, observe the skin condition,score it, (**Table 2**) and take photographs approximately every 3–4 days. Accumulate scores according to the following criteria. The dorsal skins were excised for the next experiments. The dorsal skin sections were divided into three parts on the basis of use. One part was transferred into 10% formalin for histologic analysis.

**Table 2:** Atopic Dermatitis Rating.

Atopic Dermatitis Rating	Score
Asymptomatic	0 points
Scratching	1 point
Skin breakdown/desquamation	3 points
Production of mucus and body fluids	4 points
Production of mucus and body fluids/pus/bleeding	5 points

### 2.6 Respiratory resistance test

Measure respiratory resistance (Penh (Enhanced Pause) value, maximal inspiratory volume, maximal expiratory volume, expiration time, airway resistance, and breathing rate).

### 2.7 Data analysis

Data were calculated and expressed as mean ± standard deviation or percentage. Comparisons between groups were performed via one-way ANOVA with either a *post hoc* Duncan t test analysis for comparison between all groups (SPSS version 26.0, IBM Inc.). The p values of less than 0.05 were considered as statistically significant. These analyses were accomplished by using statistical analysis system configured for computer (SPSS, Vision 26.0).

### 2.8 Materials

We utilized a TECAN Sunrise spectrophotometer and a ProCyte Dx hematology analyzer (IDEXX) for our analyses. Mouse samples were evaluated using various ELISA kits, including Total IgG (Invitrogen, Cat. No. 88-50400-22), Total IgE (Invitrogen, Cat. No. 88-50460-88), IgG1 (Invitrogen, Cat. No. 88-50410), IgG2a (Invitrogen, Cat. No. EMIGG2AX10), and OVA-specific IgE (BioLegend, LEGEND MAX™, California, USA). Additionally, cytokine levels were measured using mouse IFN-γ (Invitrogen, Cat. No. 88-7314-88) and TNF-α (Invitrogen, Cat. No. 88-7324-88) ELISA kits.

## 3 Results

### 3.1 Weekly weight changes

**Figure 1:**
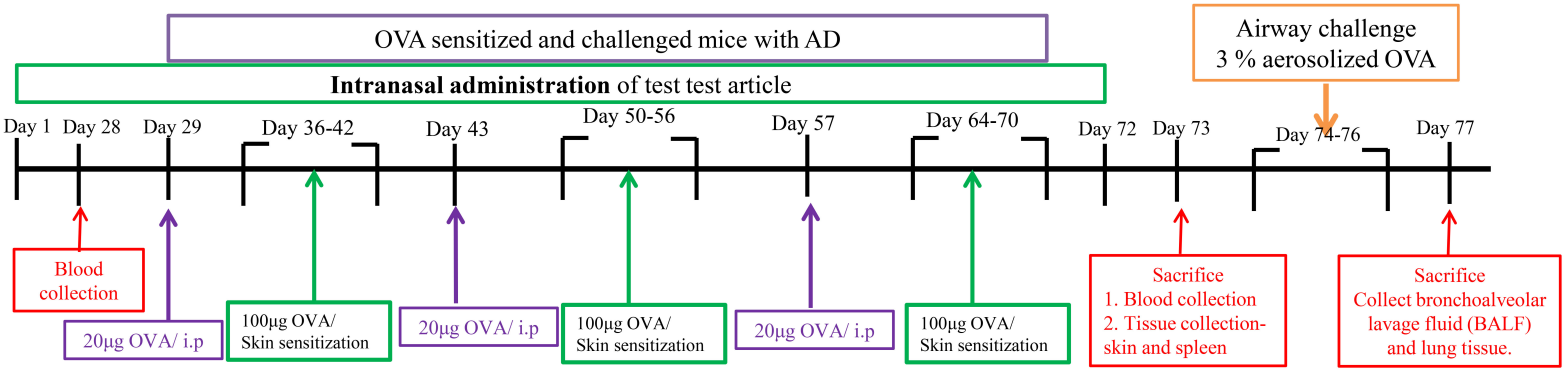
Experimental time point. After 1 week of stasis after intraperitoneal injection, OVA was used to stimulate the skin for 7 days. This is one cycle, with a total of 3 cycles. Method for inducing asthma mode in the late stage of atopic dermatitis: 73 days after AD mode was induced, asthma mode was induced with 3% OVA aerosol for 3 consecutive days.

Although there were significant differences in the initial weights among the groups before treatment (p<0.05), the weights were relatively close at 21.366 ± 0.933 g. Animals in all groups showed stable weight increases from Week 1 to Week 4. However, due to OVA stimulation in the fourth week, there was a decline in weight during Weeks 5 and 6. Following the induction of atopic dermatitis, except for the control group, animals in the other groups exhibited weight decreases. From Weeks 8 to 10, the control group showed significantly higher weights compared to the other groups (p>0.05). Detailed weight data are depicted in **Figure 1**.

### 3.2 Skin condition

**Figure 2:**
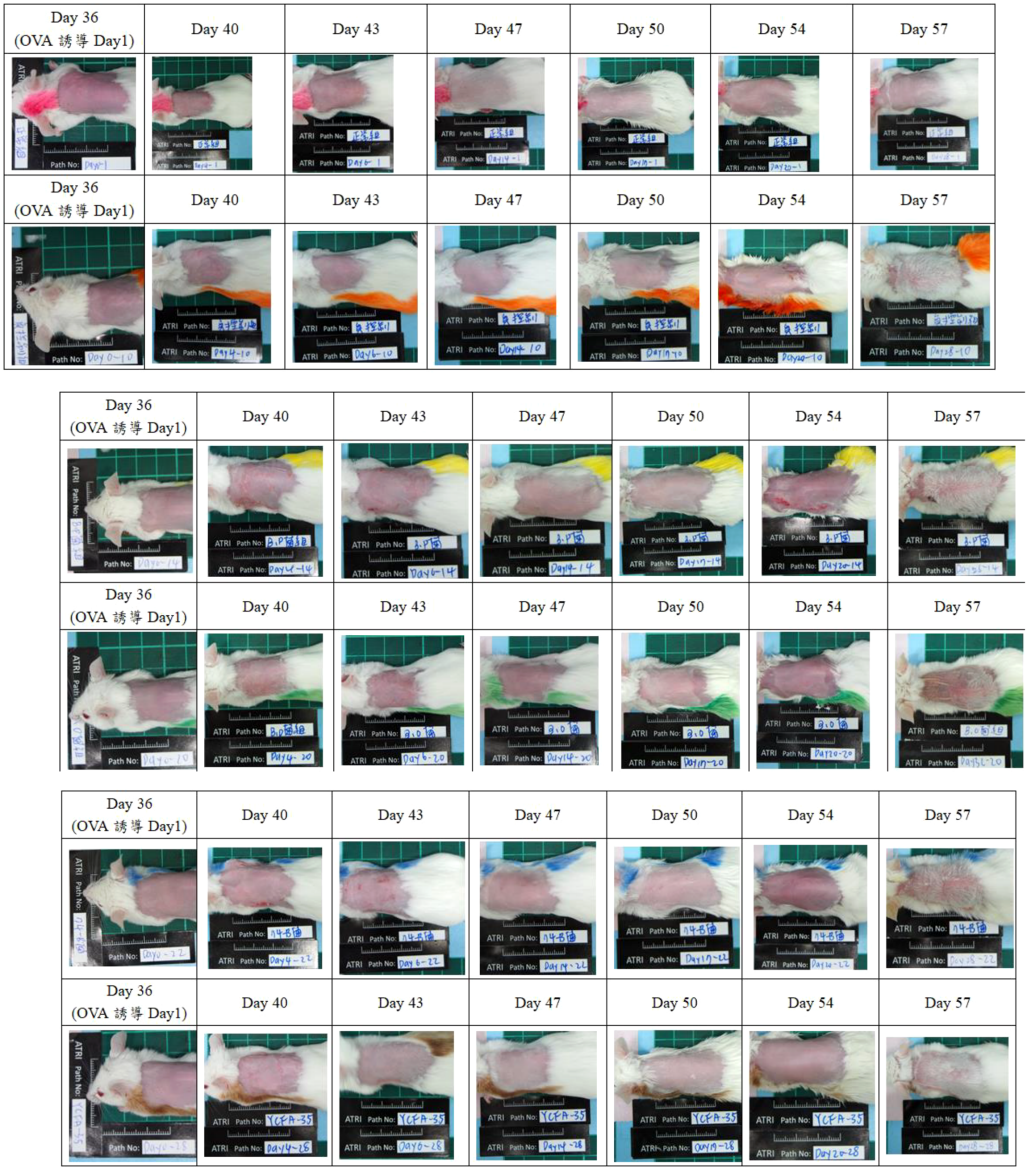
Skin condition observation. The skin status of each group before OVA stimulation was observed to be asymptomatic. The negative control group had more obvious skin oozing after OVA induction; in the b.p group, symptoms of mild skin itching and skin desquamation were even more severe after OVA induction. Obvious skin bleeding; slight skin itching after OVA induction in group b.o and even more obvious skin bleeding; skin itching/bleeding after OVA induction in group 74-B; YCFA-33 group Mild skin itching symptoms occurred after OVA induction. This result shows that the 74-B and YCFA-33 groups may alleviate the symptoms of redness, swelling and itching of the skin caused by OVA-induced atopic dermatitis.

Before OVA stimulation, the skin status of each group was asymptomatic. The negative control group had obvious skin itching and erythema on the 3rd day after OVA induction (Day 40), and more obvious skin rashes on the 20th day after OVA induction (Day 54). Slight skin itching and skin desquamation occurred on the 4th and 6th days (Day 40 and 43) after OVA induction in the B.P group, and more obvious skin bleeding occurred on the 20th day (Day 54) after OVA induction. Slight skin itching symptoms occurred on the 4th and 6th days (Day 40, 43) after OVA induction in the B.O group. See **Figure 2** for details.

**Figure 3:**
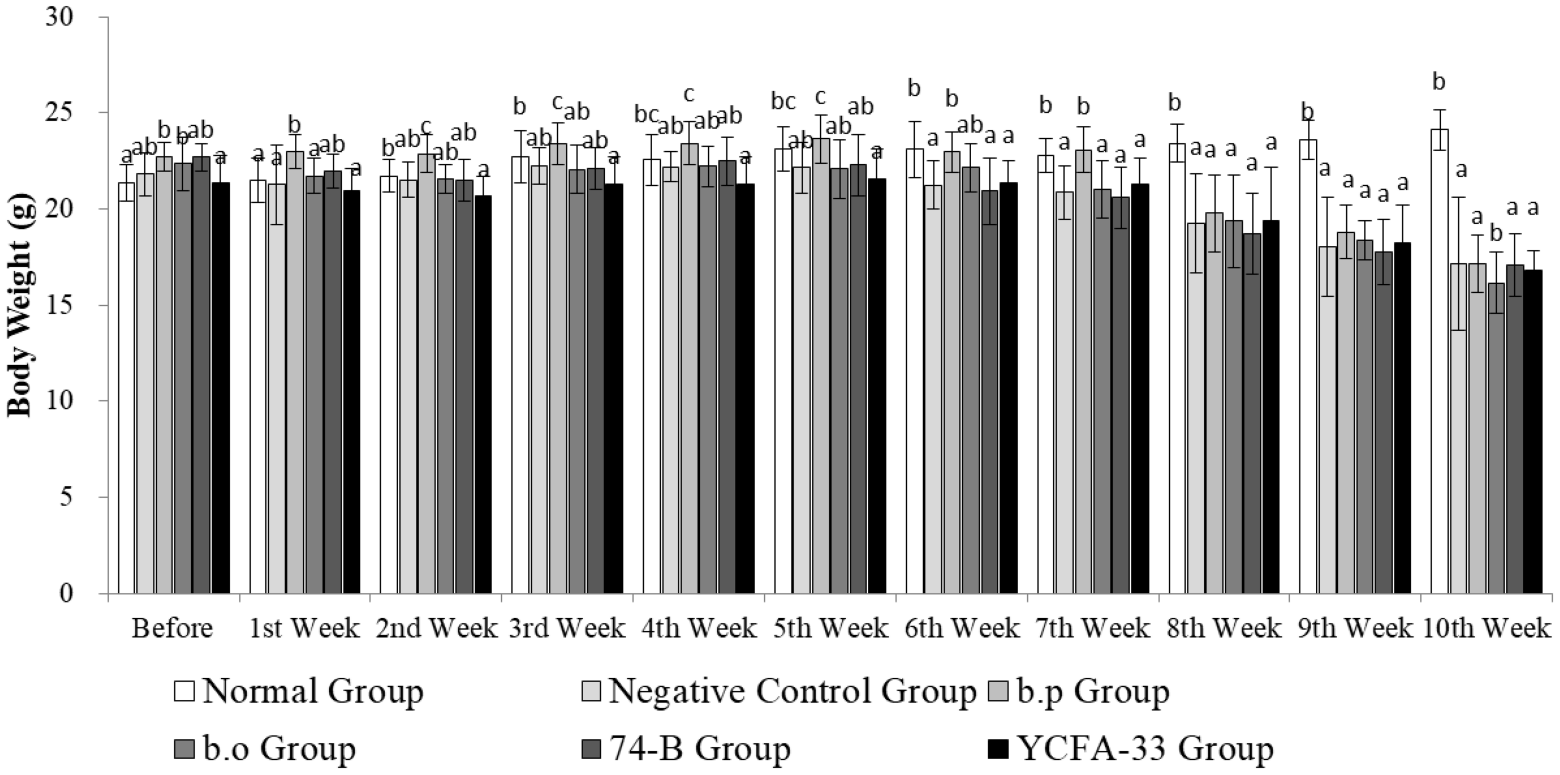
Weekly weight changes. The weight of the animals in each group increased steadily from the 1st to the 4th week. In the 4th week, the weight of the animals in the 5th and 6th weeks decreased due to the stimulation of OVA. Subsequently, the atopic dermatitis pattern was induced. The weight of the animals in the other groups except the normal group increased. From the 8th to the 10th week, the weight of the normal group was significantly higher than that of other groups (p>0.05).

In the 74B group, skin itching/bleeding occurred on the 4th and 6th days (Day 40, 43) after OVA induction. Mild skin itching symptoms occurred on the 4th day (Day 40) after OVA induction in the YCFA-33 group, as shown in **Figure 3**. These results showed that the administration of 74-B and YCFA-33 may have alleviated the symptoms of redness, swelling and itching of the skin caused by OVA-induced atopic dermatitis.

### 3.3 Respiratory resistance value (Penh Enhanced Pause, Penh)

**Figure 4:**
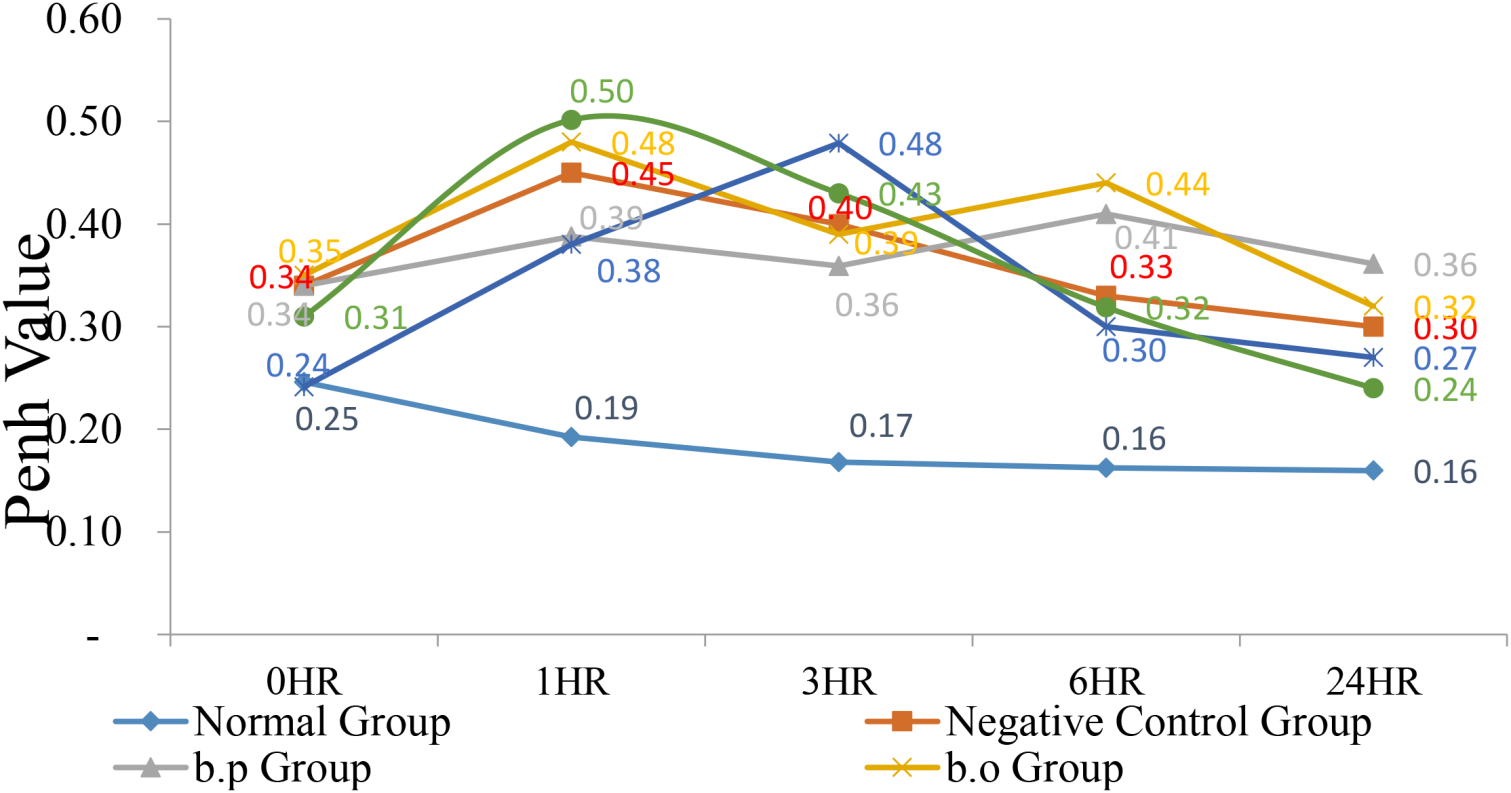
Respiratory resistance value (Penh Enhanced Pause, Penh). At 0 hr, the normal group and the 74-B group had lower Penh values. After administration of 3% OVA, the Penh values at 1, 6, and 24 hrs were monitored. At 1 hr, except for the normal group, other test groups had higher Penh values. According to the performance, the Penh value of each group showed a downward trend after 6 hr, with the 74-B group being the lowest. Administration of 74-B and YCFA-33 may reduce the respiratory tract irritation of OVA-sensitized mice. Reduce the tendency of allergic respiratory tract inflammation.

At 0 h, the normal group and the 74-B group Penh values were 0.25 and 0.24 had lower Penh values than other group. After the administration of 3% OVA, the Penh values at 1, 6, and 24 h were monitored. At 1 h, except for the normal group value was 0.19 lower than other test groups. YCFA-33 Penh value was 0. 50 higher than other group. After 6 h, the Penh values of both the 74-B and YCFA-33 groups Penh values were 0.30 and 0.32 showed a downward trend, with the 74-B group being the lowest. At 24 h, the values of each group returned to near the 0 h value. 74-B and YCFA-33 may have reduced respiratory tract irritation in the sensitized mice administered with OVA and reduced the tendency of allergic respiratory inflammation. See **Figure 4** for details.

### 3.4 Serum immunoglobulin detection and analysis

**Figure 5:**
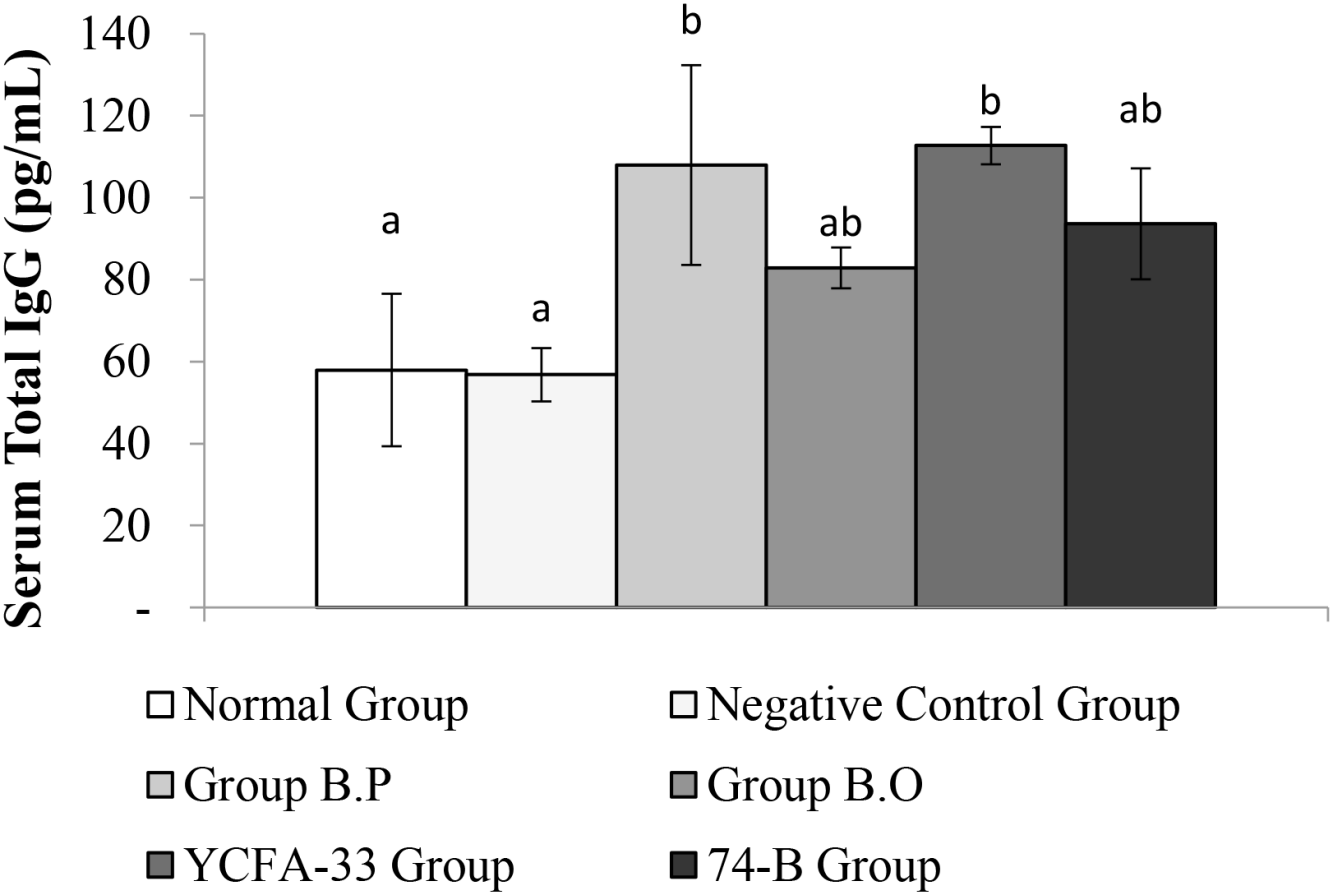
Detection and analysis of Total IgG in serum. Administration of b.p bacteria and YCFA-33 bacteria can significantly increase the Total IgG content in serum (p<0.05). Different letters ab represent significant differences (p<0.05).

**Figure 6:**
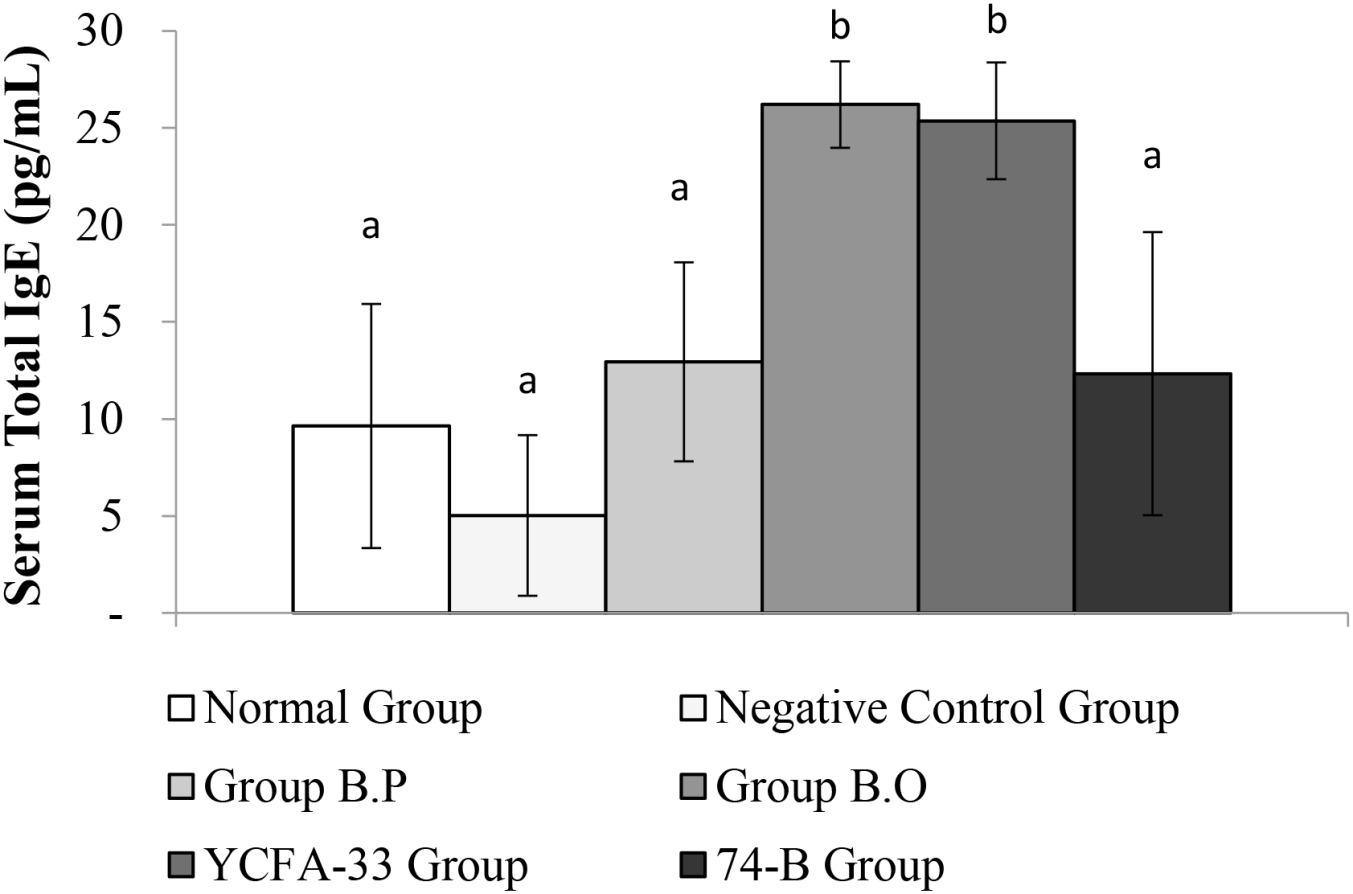
Detection and analysis of Total IgE in serum. There was no significant difference between the normal group and the negative control group. The total IgE in the serum of the b.o bacteria and YCFA-33 bacteria groups was significantly higher than that of the negative control group (p<0.05). Different letters ab represent significant differences (p<0.05).

**Figure 7:**
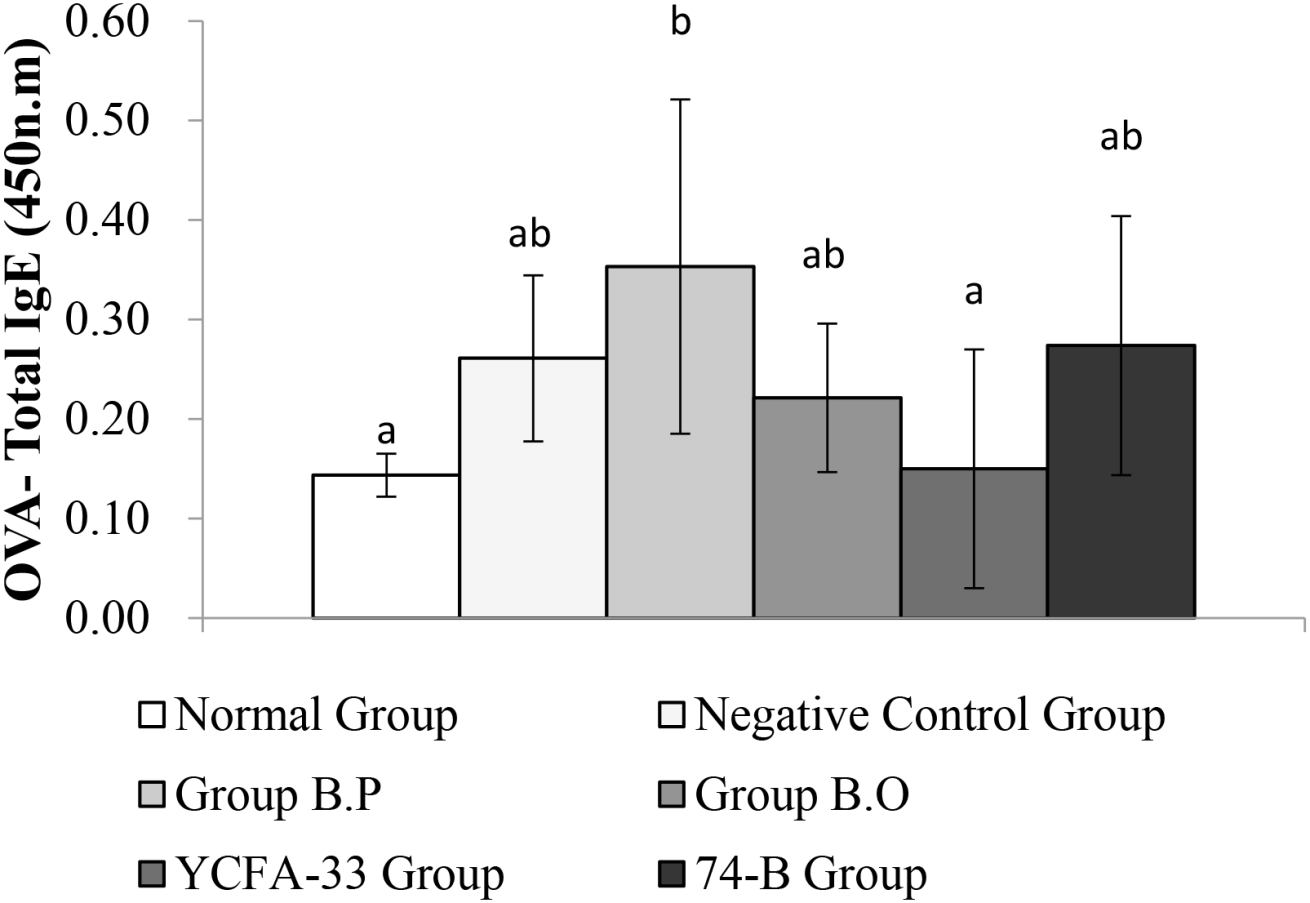
Detection and analysis of OVA-IgE in serum. There was no significant difference between the normal group and the negative control group, and there was no significant difference between the YCFA-33 bacteria group and the negative control group. However, the OVA-IgE value decreased by 43% compared with the negative control group. Different letters ab represent significant differences (p<0.05).

Total IgG in the serum of the b.p and YCFA-33 groups was significantly increased compared with the negative control group (p<0.05), suggesting that b.p and YCFA-33 could effectively increase the total IgG content in serum. See **Figure 5** for details. Total IgE in the serum of the b.o and YCFA-33 groups was significantly higher than that of the negative control group (p<0.05). There was no tendency to lower the immune response in the serum. See **Figure 6** for details. The OVA-IgE value was 43% lower in the YCFA-33 group compared with the negative control group, although the difference was not significant. This suggested that the administration of YCFA-33 may have reduced the immune inflammatory response induced by OVA in the serum. See **Figure 7** for details.

**Figure 8:**
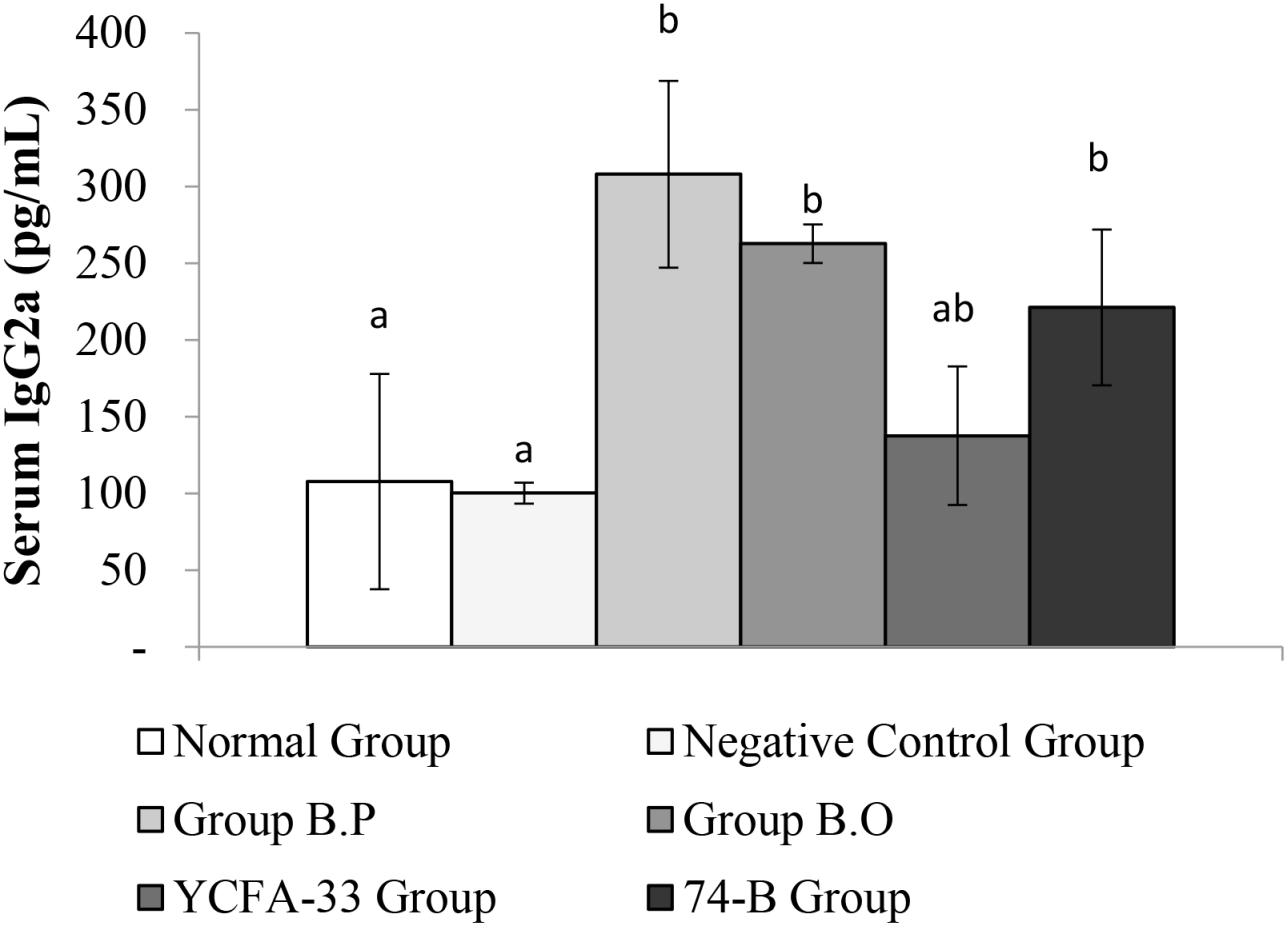
Detection and analysis of IgG2a in serum. There was no significant difference between the normal group and the negative control group. Compared with the negative control group, the serum IgG2a of B.O bacteria, YCFA-33 bacteria, and 74-B bacteria groups all increased significantly (p<0.05). Different letters ab represent significant differences (p<0.05).

**Figure 9:**
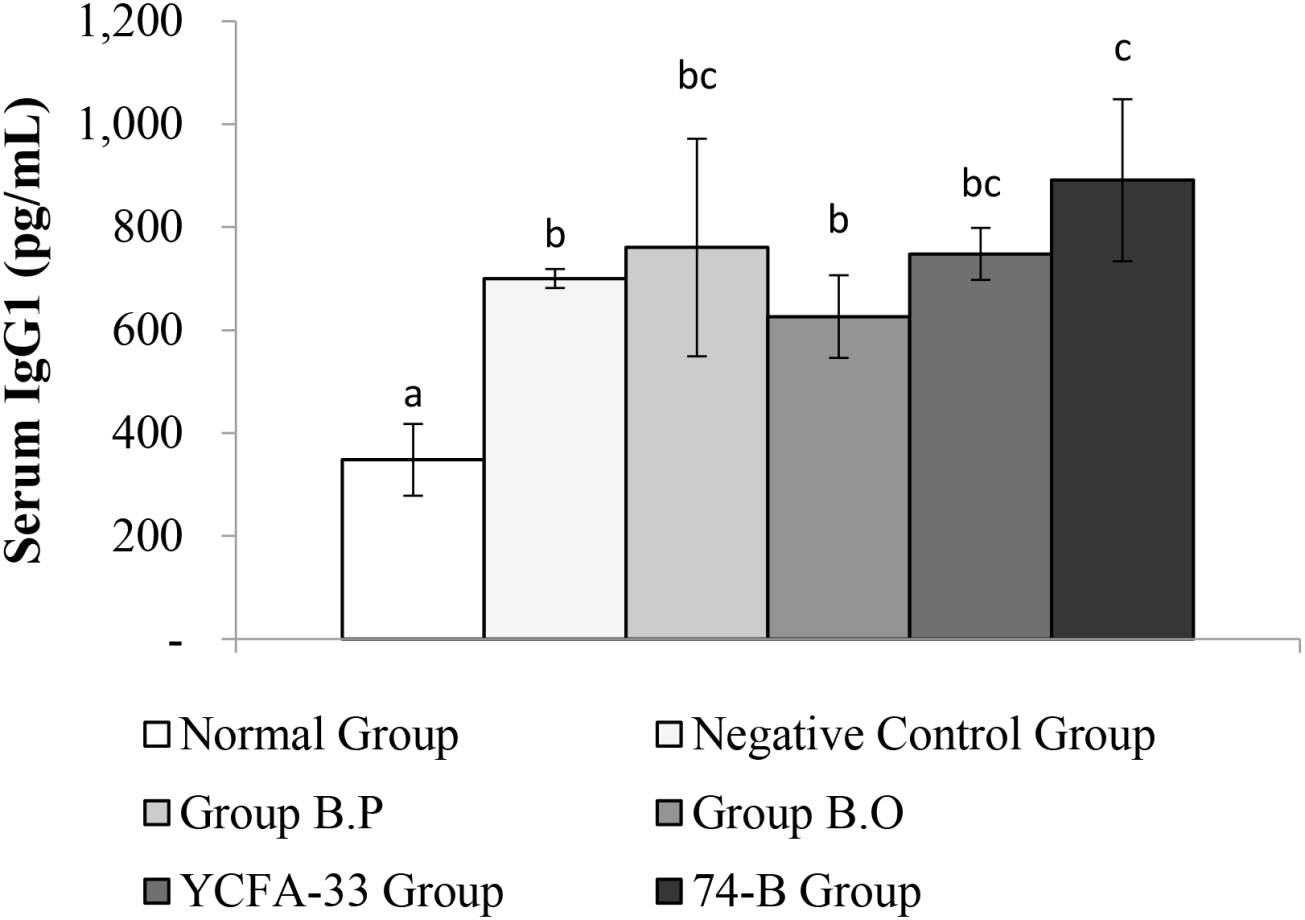
Detection and analysis of IgG1 in serum. There was a significant difference between the normal group and the negative control group (p<0.05), and there was a significant increase in serum IgG1 between the 74-B bacteria group and the negative control group (p<0.05). Different letters ab represent significant differences (p<0.05).

The b.o, YCFA-33, and 74-B groups all had a significant increase in serum IgG2a compared with the negative control group (p<0.05), suggesting that the administration of these three strains could effectively increase IgG2a in serum, subsequently improving immunity. See **Figure 8** for details. In addition, compared with the negative control group, the 74-B group had a significant increase in serum IgG1 (p<0.05). Although there was no significant difference between the b.o and YCFA-33 groups with the negative control group, the serum IgG1 levels increased respectively. The 9% and 7%, administration of 74-B bacteria could effectively increase IgG1 in serum and improve immunity. See **Figure 9** for details.

### 3.5 Types and numbers of BALF

**Figure 10:**
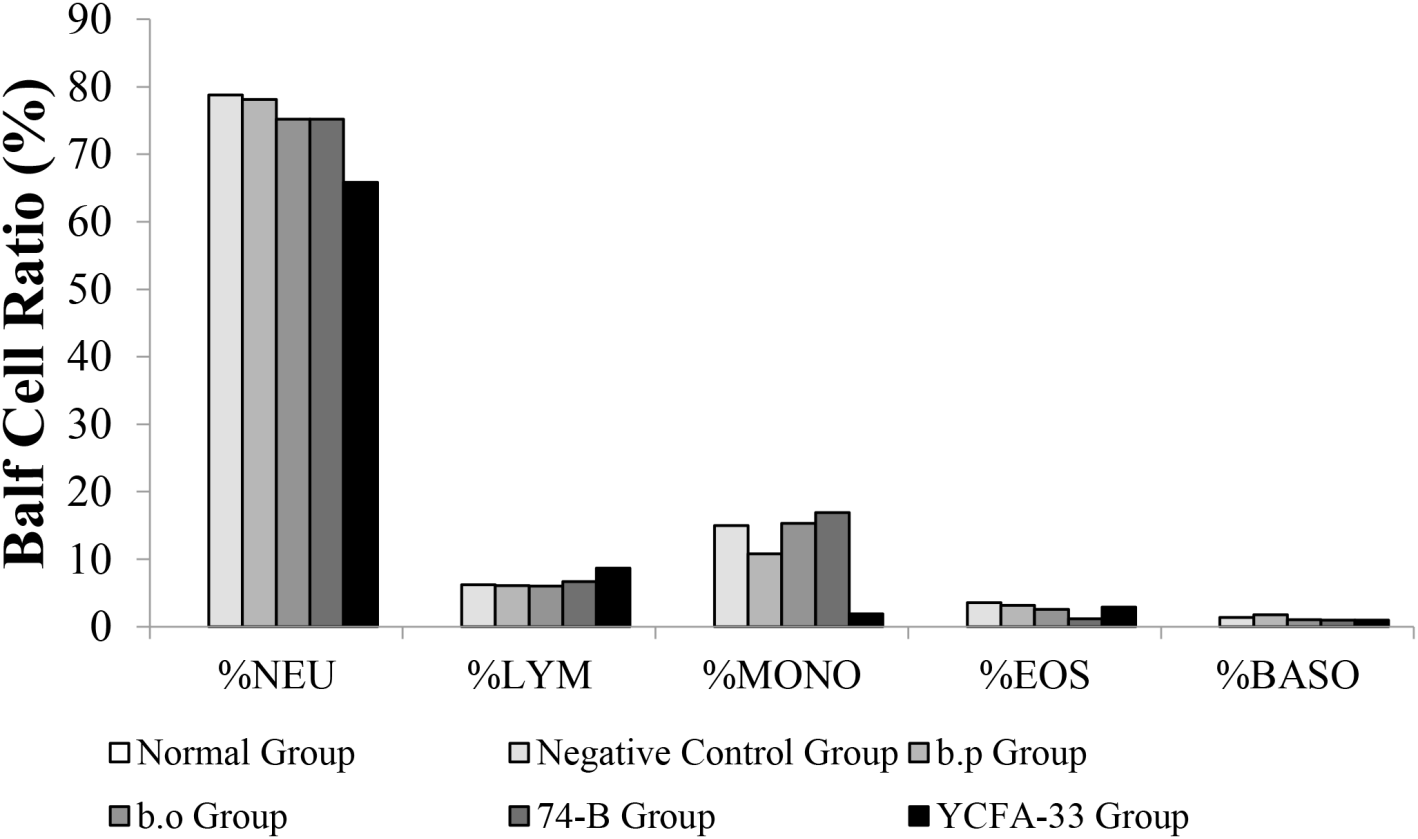
Types and numbers of lung washout cells. The content of white blood cells in the lung washing fluid of the normal group was low, and neutrophils and monocytes were not detected. However, giving YCFA-33 to white blood cells and neutrophils could reduce the number of white blood cells and neutrophils in the lung washing fluid. The number and proportion of neutrophilic pellets can effectively slow down the inflammatory response caused by OVA-induced asthma.

The number of white blood cells in the normal group was 0.28% lower than that in the negative control group value was 5.03%. The YCFA-33 group WBC value was 1.03% had a lower than the negative control group (p=0.114). See **Figure 10** for details.

The YCFA-33 group neutrophils value was 65.8% lower than negative control group value was 78.80% and the negative control group had a similar number of lymphocytes and monocytes balls to the other groups. The number of eosinophilic and basophilic spheres in each experimental group and the negative control group were similar.

### 3.6 Cytokines in BALF

**Figure 11:**
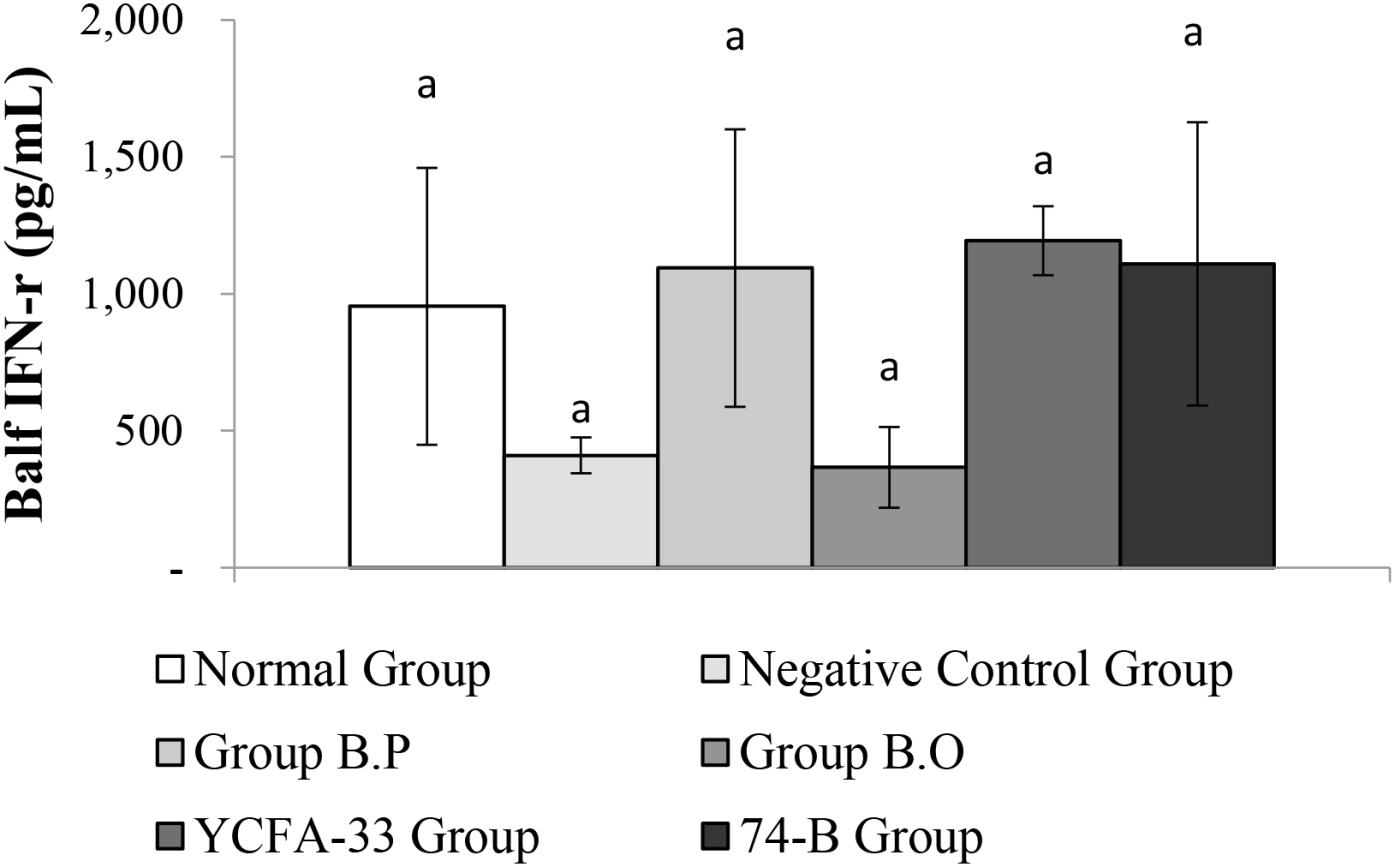
Detection and analysis of IFN-γ in lung washing fluid. There were no significant differences among the groups. aThe same letter indicates no significant difference (p>0.05).

There was no significant difference in IFN-γ between the groups. Although there were no significant differences between the b.p, YCFA-33, and 74-B groups compared with the negative control group, the IFN-γ content was 63%, 66%, and 63% higher, respectively. This suggested that the administration of b.p, YCFA-33, and 74-B could increase the IFN-γ content in lung flushing fluid, activate Th1 cells, activate killer cells, and inhibit the differentiation of Th2 cells and the synthesis of secreted IgE. See **Figure 11** for details.

**Figure 12:**
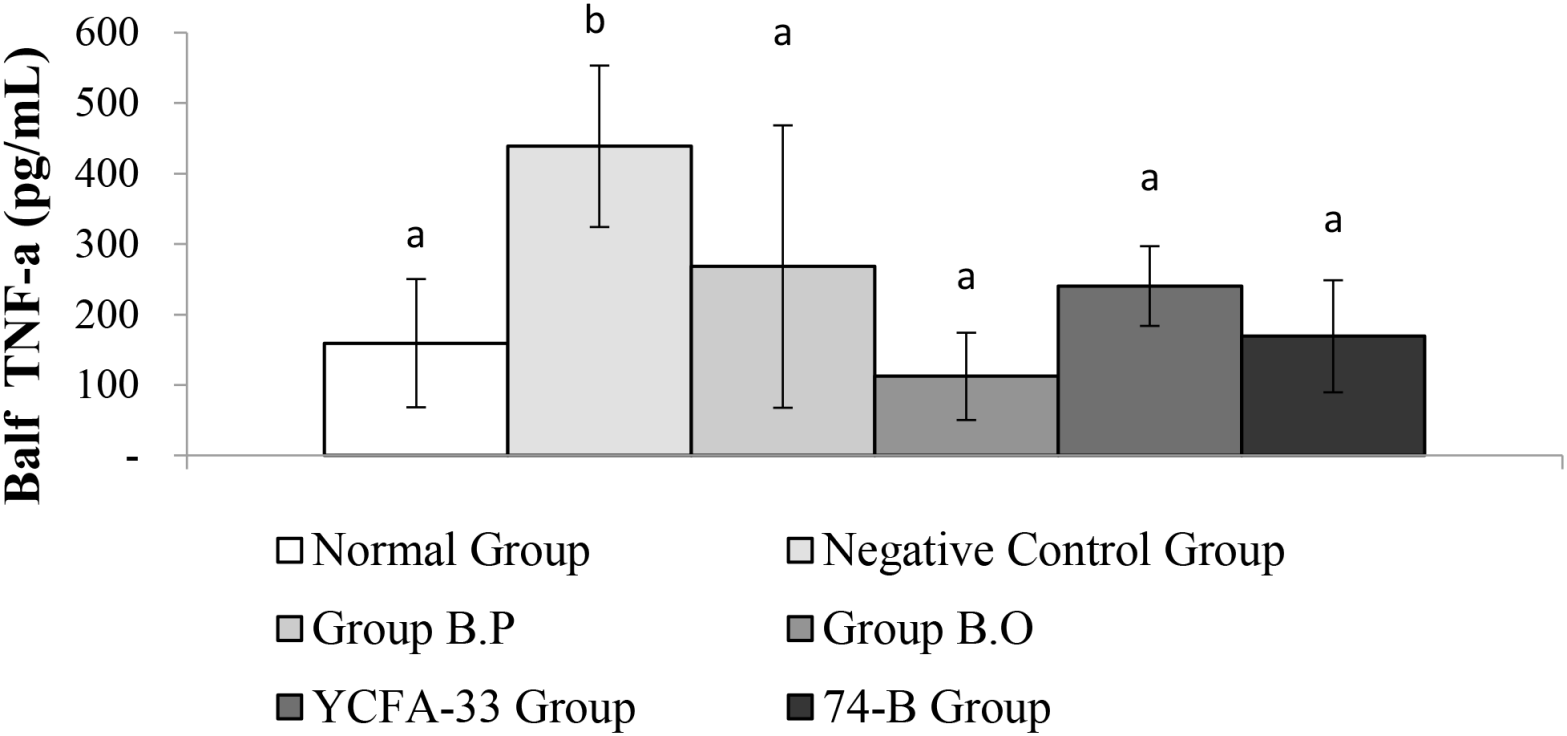
There is a significant difference between the normal group and the negative control group (p<0.05). The b.p bacteria, b.o bacteria, YCFA-33 bacteria and 74-B bacteria groups have TNF- in the lung wash fluid compared with the negative control group. The α content decreased significantly (p<0.05). Different letters ab represent significant differences (p<0.05).

There was a significant difference in TNF-α between the normal group and negative control group (p<0.05). Compared with the negative control group, the B.P, B.O, YCFA-33 and 74-B groups had similar TNF-α content in BALF. There was a significant decrease (p<0.05). These results suggested that the administration of B.P, B.O, YCFA-33 and 74-B bacteria could effectively reduce the secretion of pro-inflammatory factors such as TNF-α in BALF and reduce neutrophils. See **Figure 12** for details.

## 4 Discussion

There have been many studies on the immunomodulatory efficacy of probiotics. Animal studies on asthma patterns are also very mature. Probiotics also need more research on atopic dermatitis. For example, the use of probiotics containing four types of probiotics: Bifidobacterium, Bifidum, Bifidobacterium longum, Lactobacillus plantarum, Lactobacillus rhamnosus probiotic mixture reagent and Natto bacteria (Bacillus natto Sawamura) were tube fed to mice (6); during the experiment, 5x108 CFU/mL Lb. kefiranofaciens M1 was fed Research on AD (7, 8); The administration method for evaluating AD models is usually oral administration. In this trial, we used intranasal administration. Currently, a few studies in Taiwan use this method. method, and in the only literature results, it was found that the route of administration of probiotics is one of the most important factors affecting their efficacy, using intranasal administration and oral administration to achieve respiratory resistance, serum specific IgE/IgG2a, lung There are differences and effects on cell infiltration conditions and the performance of cytokines in BALF, which can inhibit allergic asthma symptoms, such as AHR, allergen-specific IgE, Th2 cytokine reduction, and inflammatory cell infiltration (9). The effectiveness of nasal administration of certain probiotic strains can be attributed to their ability to directly interact with the mucosal surfaces of the upper respiratory tract. Nasal administration allows these probiotics to quickly colonize the nasal mucosa, where they can modulate local immune responses and enhance the production of anti-inflammatory cytokines. This direct route of administration may be particularly beneficial in treating allergic rhinitis and asthma, as it targets the primary site of inflammation and allergic reaction more efficiently than systemic routes (10).

In contrast, oral administration of probiotics is often more effective for strains that influence the gut microbiome and systemic immunity. Upon ingestion, these probiotics colonize the gut, where they interact with the gut-associated lymphoid tissue (GALT) to modulate immune responses that can have a broader impact on allergic diseases, including atopic dermatitis and food allergies. The gut’s central role in the immune system makes oral administration a powerful method for systemic immune modulation, leading to improvements in allergic symptoms beyond the gastrointestinal tract (11).

The primary difference between inhalation or nasal administration and oral administration lies in the scope and mechanism of action (12). Inhalation and nasal routes are localized and provide direct access to the respiratory mucosa, making them particularly suitable for treating respiratory allergies. On the other hand, oral administration offers a systemic approach, where probiotics can exert their effects on multiple immune pathways through the gut, providing benefits for a wider range of allergic conditions (13).

The study’s strength is the direct comparison between oral and nasal routes of probiotic administration. This approach allows for a clear understanding of how the mode of delivery affects the immune response, providing insights into the most effective strategies for probiotic use in allergic disease management. The inclusion of both routes adds depth to the findings, potentially leading to more targeted and effective therapeutic interventions. The study’s method of evaluating serum levels of key immune markers, such as immunoglobulin E (IgE), IgG, IL-4, IFN-γ, and TNF-α, as well as conducting histological evaluations of skin and lung tissues, is a significant strength. This detailed immune profiling enables a thorough investigation of the underlying mechanisms by which probiotics exert their effects, offering a deeper understanding of the immune pathways involved in allergic diseases.

One limitation of the study is its reliance on mice as the model organism. While animal models are invaluable for preliminary research, the findings may not fully translate to humans due to species-specific differences in immune system function and responses to probiotics. This limitation highlights the need for subsequent clinical studies in humans to validate the results and determine their applicability to human allergic diseases.

The study’s design might be limited by the use of specific probiotic strains and a single dosage regimen. Different strains of probiotics can have varying effects on the immune system, and the efficacy of probiotics often depends on the dosage and frequency of administration. This limitation suggests that further research is needed to explore different strains and dosing regimens to identify the most effective combinations for managing allergic diseases. While the study provides valuable insights into the effects of different probiotic administration routes on allergic diseases, its limitations underscore the importance of cautious interpretation and the need for further research, particularly in human clinical trials and with varying probiotic strains and dosing regimens. Intranasal YCFA-33 could effectively reduce the OVA-IgE content in serum while oral route YCFA-33 could not. Intranasal route may be another choice if oral route is not effective.

The intranasal route for probiotic administration may offer superior efficacy compared to the oral route due to its direct interaction with the nasal-associated lymphoid tissue (NALT), a critical component of the mucosal immune system (14). Unlike the oral route, where probiotics must survive the harsh gastrointestinal environment, including acidic pH and digestive enzymes, intranasal delivery bypasses these barriers and enables probiotics to colonize the upper respiratory tract more efficiently (14). This targeted delivery facilitates a more rapid and localized modulation of immune responses, enhancing mucosal immunity through the production of secretory IgA and the activation of regulatory T cells (15). Additionally, the nasal mucosa provides a rich vascular network that allows for quicker systemic dissemination of probiotic-derived bioactive compounds, potentially leading to broader immunomodulatory effects (16). By directly influencing the respiratory microbiome and immune homeostasis, intranasal probiotics hold promise for preventing and managing allergic and infectious diseases with improved bioavailability and therapeutic outcomes (16).

## 5 Conclusion

In this study, we administered these four strains of bacteria via intranasal instillation to induce atopic dermatitis, and then administered OVA to the trachea to induce an asthma model to explore whether these four strains of bacteria could effectively slow the progression of AD and late-stage asthma. The results showed that YCFA-33 could effectively increase the levels of total IgG and IgG1 in serum and reduce the OVA-IgE content in serum, and that 74-B could effectively increase the level of IgG2a in serum. YCFA-33 and 74-B both increased the IFN-γ content in lung flushing fluid, activated Th1 cells, activated killer cells, inhibited Th2 cell differentiation and the synthesis of secreted IgE, and reduced the secretion of pro-inflammatory factors such as TNF-α in lung washing fluid, thereby reducing the infiltration of neutrophils and other inflammatory cells. In addition, 74-B and YCFA-33 could reduce respiratory tract irritation in the mice sensitized by OVA and reduce the tendency of allergic respiratory inflammation. Moreover, it could potentially alleviate the symptoms of redness, swelling and itching of the skin caused by OVA-induced atopic dermatitis.

## Data Availability

The original contributions presented in the study are included in the article/supplementary material. Further inquiries can be directed to the corresponding author.
